# Collegiate Year for Medical Matriculants

**Published:** 1913-07

**Authors:** 


### BUFFALO MEDICAL JOURNAL

**A Monthly Review of Medicine and Surgery**

EDITOR AND PUBLISHER DR. A. L. BENEDICT, 228 Summer St., orner of Elmwood Ave., Buffalo. (Address for all communications. Please make cpersonal and telephone calls before 1 P. M.)

THE BUFFALO MEDICAL JOURNAL is the third, in age, in the western contin ent. It is independent but supports professional organization. As a scientific medium it is unrestricted in territory. As a news medium. it is limited mainly to the western four districts of the Medical Society of the State of New York. The co-operation of physicians is asked in securing personal, obituary and other news items. Secretaries of Medical Societies in this region are requested to send brief reports of meetings. Full transactions will be published at cost of composition.

Subscription $2.00 a year in advance; $3.00 for two years in advance. Changes and errors in address or failure or duplication of delivery, should be reported immediately. Back numbers will be supplied if possible but requests for copies should be made promptly,

YEARLY VOLUME 68. JULY, 1913 No. 12

*Every excessive fee is an argument for the establishment of the British system of co-operative medical insurance; every inadequate fee, a basis for compensation under such a plan.*

### Collegiate Year for Medical Matriculants.

Some time ago we noted that the Medical Department of the University of Buffalo had taken the initiative toward securing the legal requirement of a year in college, in addition to the full high school course which is the present standard for medical matriculants for New York. We have also called attention to the fact that several states already require one or two years of college study and that the prestige of the Empire State must suffer if its authorities did not respond to the demand, for at least as high a standard as is required by states of less population, less wealth, and presumably less progress from the pioneer period, which excuses some degree of laxity in formal educational requirements.

We feel a sense of local and personal pride in giving publicity to the announcement made at the recent commencement season, that the University of Buffalo, without waiting for the action of the State Board of Regents, will, of its own volition, enforce the requirement of a collegiate year, beginning with the session of 1914-15. Inasmuch as the University itself made the first formal, institutional demand for such a standard—not to mention the fact that various of its alumni and faculty have individually advocated such a standard for many years—it is obvious that, to have waited for legislation, would not have indicated yielding to compulsion. Nevertheless, to have anticipated the legal requirement indicates a considerable degree of moral courage, since the action concedes to other schools a material competitive advantage, unless they also manifest the same broadminded and farseeing policy of disregarding quantity for quality.

Several medical schools, heavily endowed or liberally supported by public or private funds, spend annually more than three times the receipts from students' fees, even including institutions whose student body is large enough for the maximum economic efficiency. It is fortunate that they can do so, although it has occurred to us that the unnecessary elegance of grounds and buildings and the almost entire freedom from economic worry in the provisions for every detail, whether essential or merely convenient and desirable, surround the medical student with an atmosphere of luxury which may not be the best preparation for the extremely rarified atmosphere in which he must spend at least the first decade of life as a practitioner, unless he is himself endowed.

In contrast to such ease of management, the conduct of a practically unendowed medical school, handicapped at the outset by a somewhat limited territory and population, imposing upon itself the further restriction of standards of education surpassed in only a half dozen states and by only a few wealthy institutions, and maintaining except in unessential details the maximum standards of technical pedagogy, is a business problem demanding a grade of business sagacity and ability worthy to rank with some of the greatest exploits of the commercial world.

Right here we wish to anticipate a possible charge of inconsistency. We have, personally and editorially, since and long before assuming charge of this journal, called attention to the well-known fact that the medical profession is overcrowded, and have advocated various measures tending to re-establish a balance between supply and demand. Is it not obvious that the quickest way to bring about this end would be to close the medical colleges, using every means in our power to eliminate one after another, as they showed signs of weakness, financial or otherwise? In our opinion a prompter, more radical method and one involving exactly the same ethical factors would be the assassination of physicians already licensed. In other words, we believe in the sixth commandment and, while recognizing the ultimate economic disturbance of supply and demand, we believe that the problems which it presents must be met in a reasonable and ethical spirit. Among other reasons for avoiding harsh and radical measures are the rights of the individual to choose his own vocation, subject to reasonable limitations applicable to all, and to be able to pursue that vocation without the prohibitive requirement of study at too great a distance from home; the right of the community to continue established institutions which redound to its credit and which will become more and more necessary as time passes, after temporary inequalities of the law of supply and demand have righted themselves; the right of the personnel of the institution to continue activities for which they have fitted themselves.

A very significant factor in the present agitation with regard to the status of medical schools is the prominence given to money in applying the general principle of the survival of the fittest. Without underestimating the necessity of equipment and forms of labor requiring money, it seems to us that the utmost caution should be observed by the profession in supplanting ethical ideals with the coercive argument borrowed from certain forms of commercial enterprise.

We bespeak, therefore, for the University of Buffalo the cordial support of the profession, especially at this time when it has voluntarily assumed an additional burden in the cause of advanced educational standards. This support can be manifested in many ways, not forgetting the direct one which is so obvious as to require no mention. We trust that civic and sectional pride will also be touched by a realization that an advance in the education of the prospective medical practitioner confers an advantage on the community as well as the profession—perhaps more.

We are pleased to announce further that the University of Buffalo will, itself, organize a course including such collegiate studies as are especially preliminary to the medical course, and we hope that this course may prove to be the practical beginning of the department of liberal arts so long contemplated and so much desired.

For the present the course will be given in the Medical Building. The science course required of medical students is one year of work in Physics, Chemistry and Biology, and either German or French. There is no reason why this course may not be pursued by others than those planning to take up the study of medicine, and if a sufficient number desire it, additional courses in English and Mathematics will be given. By substituting the latter subjects for one or two of the science subjects the equivalent of the freshman year in a course leading to the degree of B. S. in standard colleges may be secured.

